# A Rare Cause of Anterior Knee Pain in a Young Athlete and a Delayed Diagnosis: Osteoid Osteoma of the Patella

**DOI:** 10.7759/cureus.6420

**Published:** 2019-12-19

**Authors:** Eftychios Papagrigorakis, Ioannis S Benetos, Matthaios Bakalakos, Meletis Rozis, Spiros Pneumaticos

**Affiliations:** 1 Orthopaedics, KAT Trauma Hospital, Athens, GRC; 2 Orthopaedics, University of Athens, KAT Trauma Hospital, Athens, GRC; 3 Orthopaedics, National and Kapodistrian University of Athens, KAT Hospital, Athens, GRC

**Keywords:** osteoid osteoma, patella, anterior knee pain, athlete

## Abstract

Intra-articular osteoid osteomas are a rare cause of articular pain. Their diagnosis can be challenging due to their non-specific clinical presentation and imaging characteristics. We present the case of a young soccer player with a 12-month history of anterior knee pain that was first attributed to Sinding Larsen Johansson syndrome and then to Hoffa’s syndrome. A CT scan was performed that revealed the localization of an osteoid osteoma of the patella. The patient was successfully treated by percutaneous radiofrequency ablation under CT guidance without complications and returned to full sports activity. Although a rare entity, osteoid osteoma of the patella with its atypical clinical features could be included in the differential diagnosis of persistent anterior knee pain in the young adult. High clinical suspicion is necessary to avoid delay in diagnosis and irrelevant procedures for the patient.

## Introduction

Osteoid osteoma (OO) is a benign bone lesion that constitutes 11% to 14% of all benign bone tumors and presents clinically with pain typically worsened at night, alleviated by nonsteroidal anti-inflammatory drugs (NSAIDs) or aspirin. The lesion is commonly found on the diaphysis or metaphysis of long bones, and its typical radiological appearance is a radiolucent zone surrounded by sclerotic bone (nidus) smaller than 1.5 cm in diameter [1].

Intra- and juxta-articular OO are a diagnostic challenge for the orthopedist due to their rare appearance (13% of the lesions) and also their atypical clinical and radiological characteristics [2]. Misdiagnosis and delay till definitive treatment is a common problem, especially when the lesions arise in a subchondral location in the knee or the patellofemoral joint [3].

We present the case of a patellar OO in a young soccer player that was treated by percutaneous ablation. The uncommon site in combination with the atypical clinical presentation caused a 13-month interval between the onset of the symptoms and final treatment.

The aim of this paper is to report this rare case of patellar OO presenting as anterior knee pain (AKP) along with a review of the literature. The difficulties a clinician faces, in his effort to diagnose a rare entity presenting with a vague knee symptomatology and thus focus on crucial points in the diagnosis of intra- and juxta-articular OO, should be highlighted.

## Case presentation

**Figure 1:**
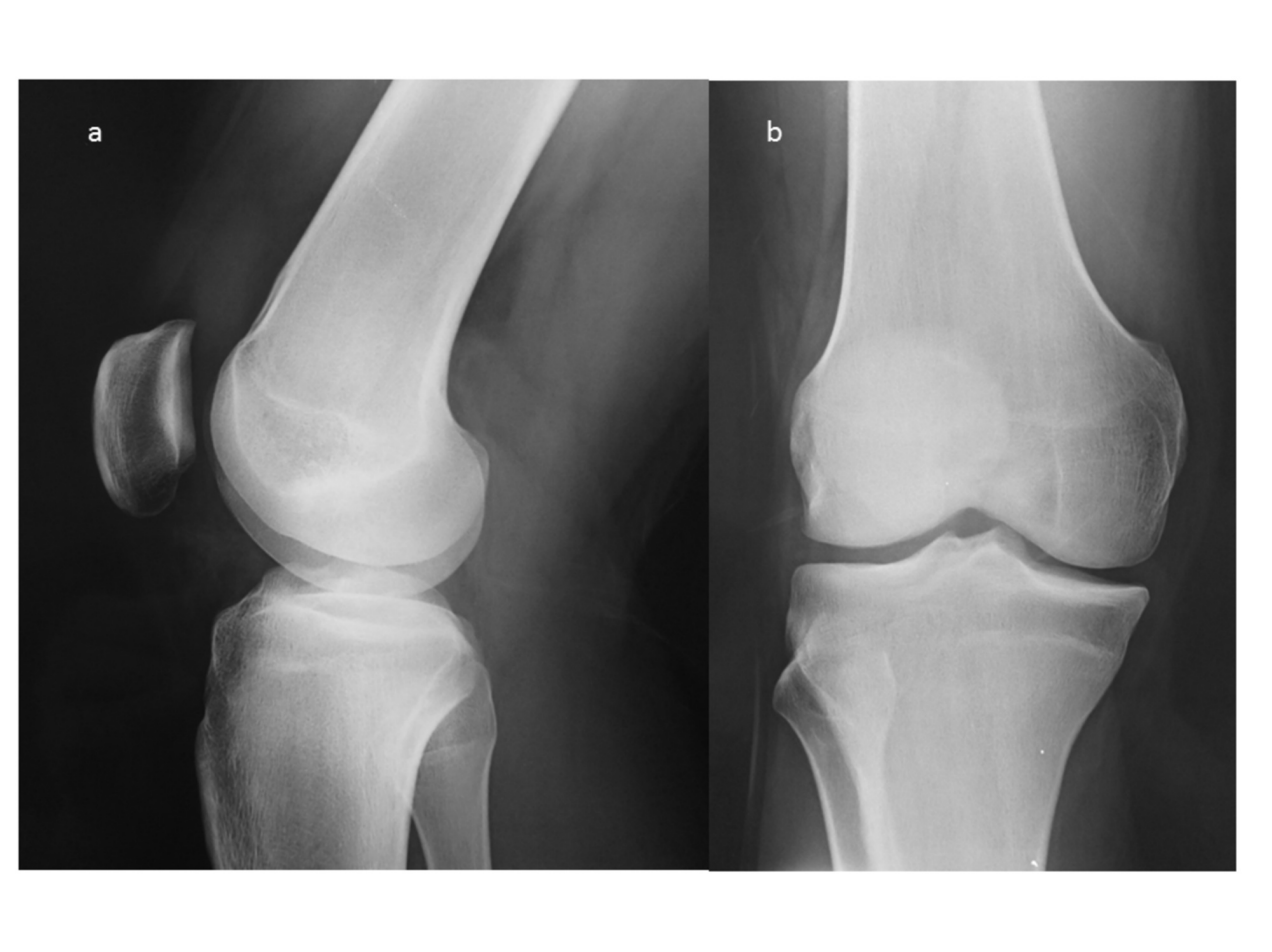
Knee x-ray of the patient a. Sagittal view, b. Coronal view

**Figure 2:**
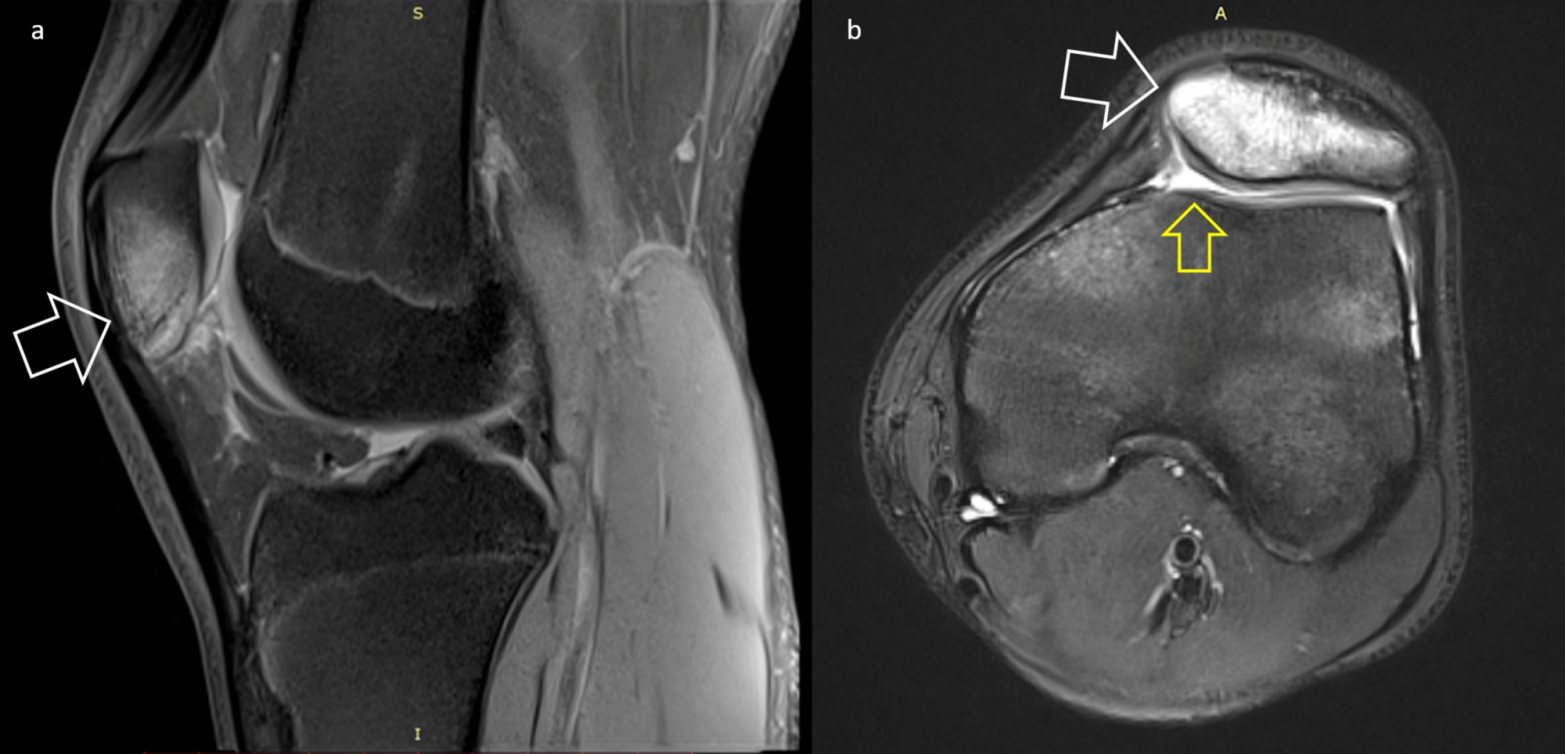
First MRI scan showing diffuse signal uptake of the patella and effusion a. Sagittal view, b. Transverse view White arrows: diffuse signal uptake, Yellow arrow: knee effusion

A 17-year-old adolescent soccer player presented with a 12-month history of left AKP. The pain was initially mild and poorly localized around the knee joint and had been attributed to a direct blow on the patella he had sustained during training. However, there was no relief with conservative treatment and physiotherapy and the pain progressively deteriorated over months. The pain was present at day and night and partially relieved with NSAIDs. An x-ray of the knee had been obtained with unremarkable findings (Figure 1). Two MRI scans had been performed with a six-month interval, both showing diffuse bone edema over the patella and mild effusion without any specific finding (Figure 2). Sinding Larsen Johansson syndrome was initially suspected, and sports participation was restricted. As the complaints became more intense, the patient stopped athletic activities and after the second MRI scan, Hoffa's syndrome was diagnosed.

**Figure 3:**
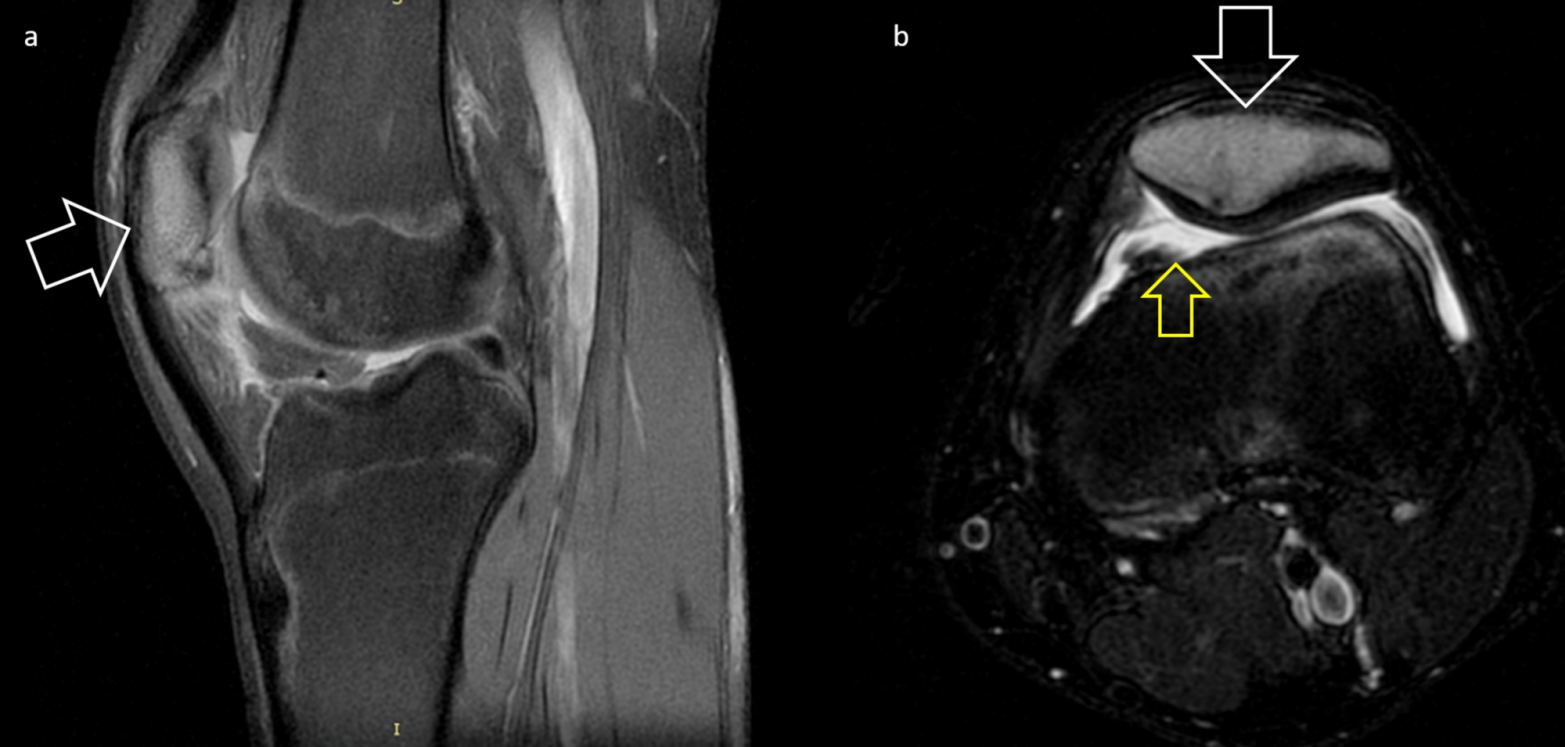
Third MRI scan, showing only non-specific findings, increased signal on T2 sequence, and mild effusion a. Sagittal view, b. Transverse view White arrows: increased signal on T2 sequences, Yellow arrow: knee effusion T2: T2-weighted image

The pain gradually worsened over the next months, and the patient was referred to our institution for further treatment. At clinical examination, serious sensitivity was noted on the patella and patellofemoral joint without any redness or local temperature rise. Range of motion was limited due to intense pain, and was aggravated by patellar squeezing. Thigh atrophy was also obvious. Laboratory studies were unremarkable. A third MRI was performed showing diffuse signal uptake on the patella and joint effusion (Figure 3).

**Figure 4:**
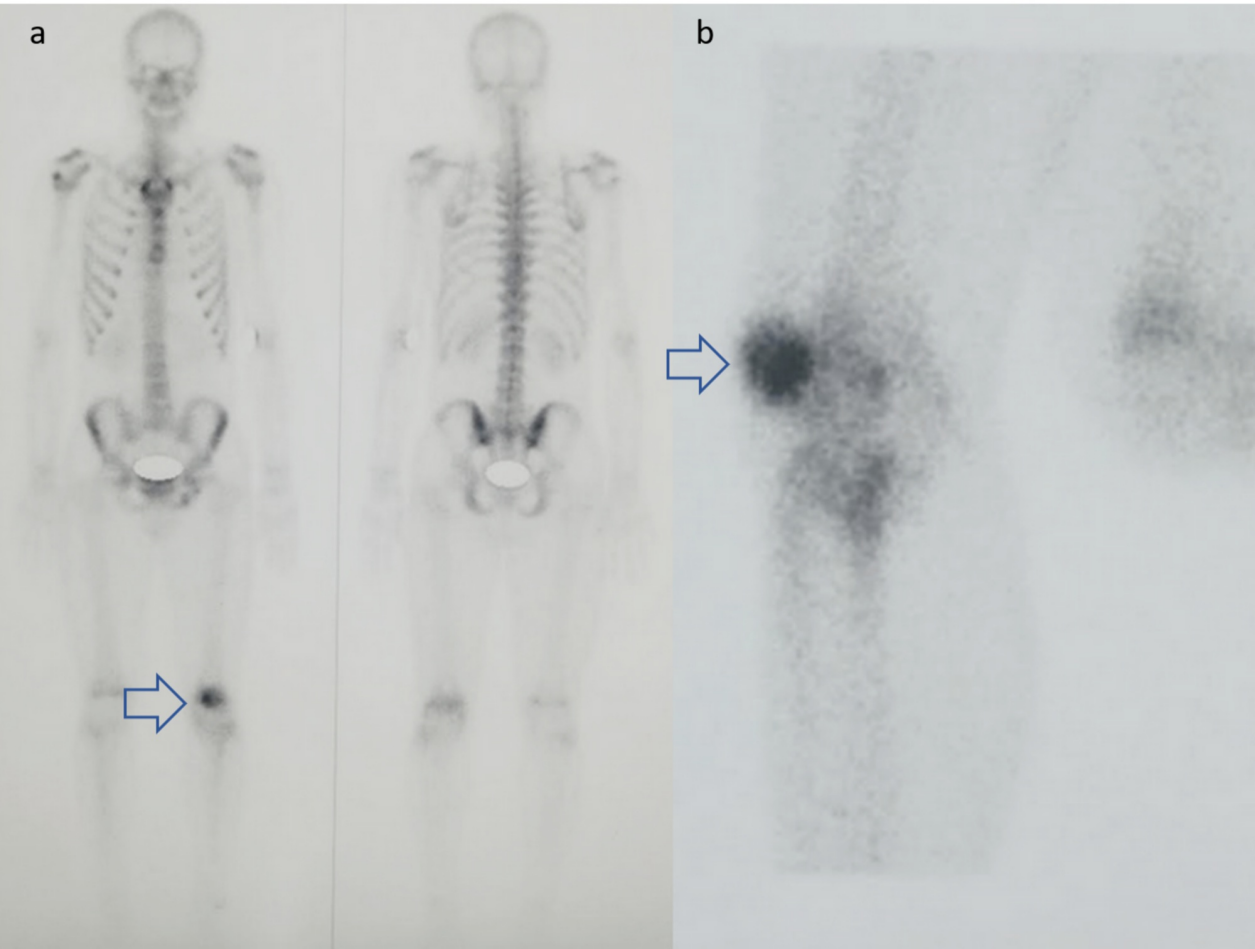
Bone scintigraphy a. Total body coronal view, b. Patella sagittal view Blue arrows: increased signal uptake on the patella

**Figure 5:**
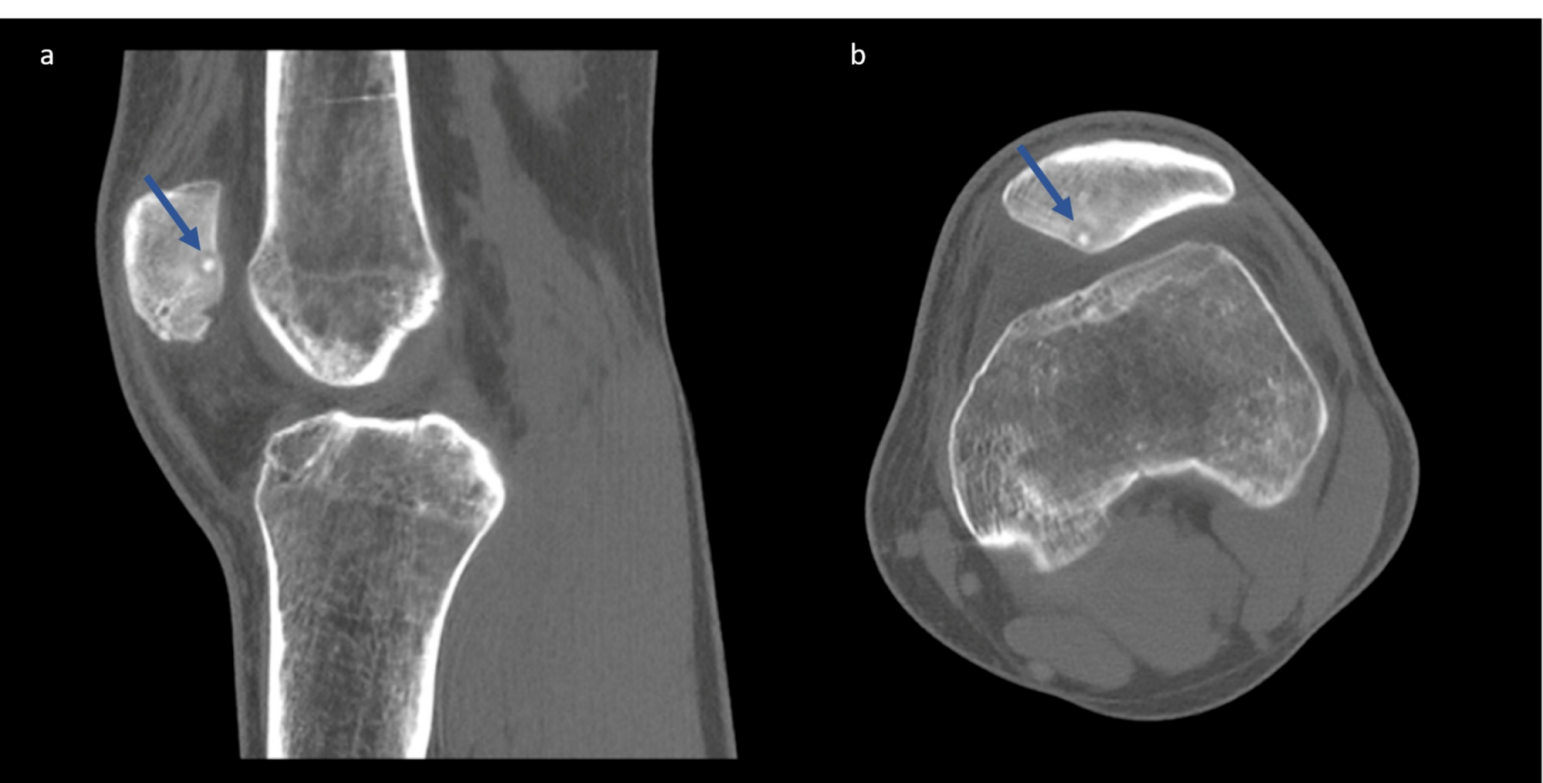
CT scan reveals the pathognomonic nidus appearance Blue arrows: characteristic nidus on a subchondral location

A diagnostic arthroscopy was scheduled. Before that a bone scan was performed, which showed intense focal uptake over the left patella (Figure 4). A CT scan followed revealing the classic nidus of an intra-articular patella OO, 12 months after the first symptoms (Figure 5). Because of the pathognomonic appearance of the nidus on the CT scan along with the results of the bone scan, the arthroscopy was cancelled and a CT-guided radiofrequency (RF) ablation was scheduled.

**Figure 6:**
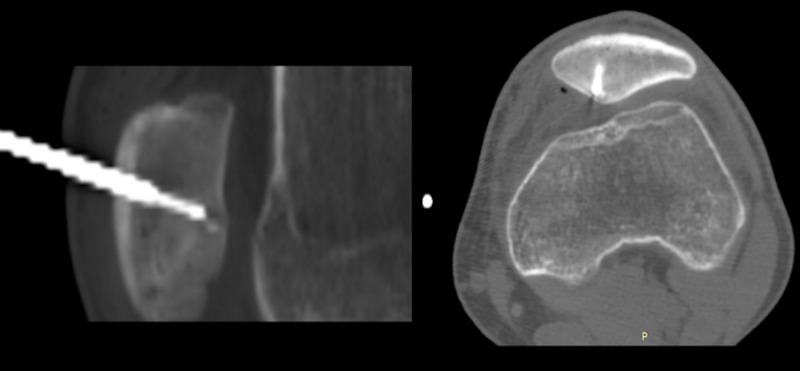
CT-guided RF ablation The needle tip (white), as it approaches the nidus RF: radiofrequency

The following month, a CT-guided RF ablation was performed. Prophylactic antibiotic treatment was administered one hour prior to the procedure. Under femoral block, without the use of a tourniquet, a small portal through the skin and the subcutaneous tissue was created with a no 11 scalpel on the anterior aspect of the patella. Through it, (avoiding any transarticular approach) a biopsy needle was transosseously inserted under CT guidance and a bony sample was obtained from the nidus. The tip of the RF electrode was precisely placed in the nidus (Figure 6), keeping a distance of 2 mm from the cartilage and RF ablation was performed.

**Figure 7:**
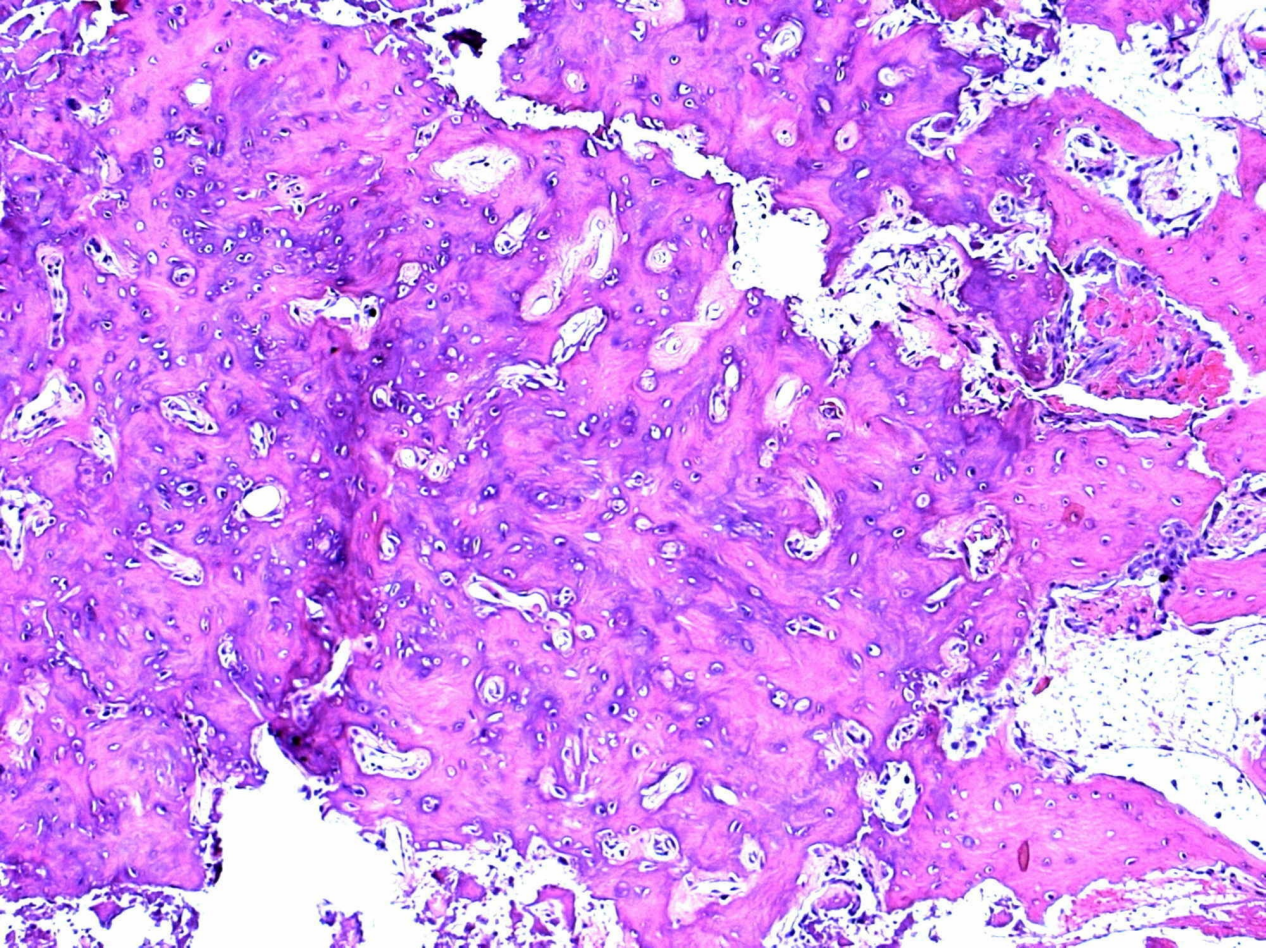
The classic appearance of the nidus of the osteoid osteoma composed of bony trabeculae, separated by vascular fibrous connective tissue

Histological study confirmed the diagnosis of OO, with the classic appearance of mineralized woven bone with regularly shaped nuclei containing little chromatin but abundant cytoplasm (Figure 7).

The patient remained hospitalized overnight and was discharged the day after the procedure with a mild discomfort on the ablation site, with full range of motion. He was advised to use crutches for the first two weeks and then proceed to full weight bearing. He returned to full sports activity one month after the procedure without any complaints. On his regular follow-up 1, 6, and 24 months following the ablation, he remained completely free of pain and satisfied with his sports performance.

## Discussion

The unique imaging features of intra-articular OO combined with their common clinical presentation make them a diagnostic challenge for the clinician. Kattapuram et al. suggested that they should be considered separate clinical entities because of the difficulties encountered in their recognition [4]. Especially around the knee joint the diagnosis can be delayed for many months [3]. In our case, the interval between the first symptoms and diagnosis was 12 months.

The first arduousness is that OO are a rare cause of joint pain. Approximately 5.2% to 13% of the OO appear in a juxta- or intra-articular position [5,6]. Hip joint is the most commonly affected area, followed by the ankle, elbow, wrist, and knee [2,6]. Patella is a rare site for OO, and only a few cases have been reported in the literature [7-13].

The most common symptom when an OO arises around the knee joint is AKP. However, AKP is one of the most frequently met musculoskeletal disorders. Each year many young athletes seen in primary care setting complain of some degree of knee pain, which is usually attributed to chondromalacia patella, patellar tendinitis, mediopatellar plica syndrome, Hoffa's syndrome, patellofemoral malalignment, osteochondritis dissecans, meniscal tears, or ligamentous injuries [14]. The pain encountered from an OO is the result of the high prostaglandin levels produced within the nidus [15]. The transmission of these prostaglandins from the nidus to synovium causes lymphofollicular synovitis, resembling histologically rheumatoid arthritis and clinically monoarthritis of infectious, degenerative, or rheumatologic origin [16]. In some cases of intraarticular OO as the one presented here, relief with salicylates or NSAIDs may be partial and typical nocturnal pain absent. The diffuse pain due to synovitis and the lesion itself accompanied by non-specific symptoms as muscle atrophy or muscle spasm around the joint, limited range of motion, joint effusion and swelling, gait and postural disturbances may be misleading for the clinician [2].

Imaging features of the intra-articular OO differ from the characteristics of the extra-articular OO. X rays are not diagnostic in 80% of the cases as at the time of the first symptoms no nidus or periosteal reaction is usually present [3,4,17]. On the other hand, intracapsular periosteum seems less able to produce thick new bone because of the absence of cambium layer and thus periosteal reaction surrounding the nidus may be minimal or absent when the lesion is located within 1 cm from the articular surface [2,4]. Another common radiological finding is juxta-articular osteopenia or osteoarthritic changes [2,18]. In scintigraphy the typical double density sign is usually absent showing diffuse uptake and generalized involvement of the joint (as in our case, Figure 4) decreasing the specificity of the examination [6,16-18].

MRI is often requested as first-line imaging when dealing with knee symptoms. In our case, three MRI scans were performed in 12 months, none of which revealed the lesion, showing diffuse bone edema over the patella and joint effusion. In the literature, it is reported that approximately 21% of intra-articular niduses are not identified and a further 29% poorly identified, on initial MRI [6]. The increased signal in some cases may even “transform” a benign lesion into an aggressive lesion and the picture can be mistaken by the radiologist as a sarcoma of bone [18]. CT scans are still considered the gold standard for detecting and diagnosing OO both in adults and pediatric population [18]. For our patient, CT proved more accurate than MRI in detection of the OO, giving us the characteristic nidus demonstration (Figure 5).

Newer techniques have shown promising results in the imaging of intra-articular OO. Especially the use of single photon emission computed tomography/CT and MRI gadolinium-enhanced imaging have proved to be efficient in detection of the nidus in intra-articular lesions, which may have been the reason for a relatively early diagnosis [5,16]. According to Szendroi et al. a careful diagnostic approach with the simultaneous use of several kinds of imaging makes it possible to confirm the diagnosis in 80% of cases of intra-articular OO before planning treatment, especially in the knee [17].

The average delay for diagnosis of intra-articular OO has been reported by Szendroi et al. at 26.6 months and by Rolvien et al. at 20.7 months [5,17]. For patellar OO, except for a case in a 13-year-old girl where diagnosis came six months after the first symptoms, the delay till diagnosis ranges from 12 months (in our case) to three years [9,12].

The aforementioned imaging difficulties accompanied by the persistent non-specific symptoms of the patellar OO can result in misdiagnosis, irrelevant surgical procedures, and great discomfort for the patient. In our case, three MRI scans and physiotherapies were performed and an arthroscopy was scheduled. Our patient presented with an ill-defined AKP that was first attributed to a mild injury during training and after that to Sinding Larsen Johansson and Hoffa's syndrome before final diagnosis. Other common misdiagnosis for patella OO across the literature includes chondromalacia patella in three cases: patellofemoral malalignment, osteochondritis dissecans, meniscal tear, and quadricept tendinitis [7-9,12,13]. Half of the cases underwent at least one diagnostic arthroscopy before final diagnosis [8,10,13]. Bavaneh et al. described a patellar OO case where the patient had three MRI scans, one diagnostic arthroscopy followed by synovial biopsy and a psychiatric consultation before the CT scan that revealed the nidus, and final treatment with open resection and mosaicplasty [13]. Cohen et al. reported a patella OO case where two diagnostic arthroscopies and a femoral biopsy were performed before diagnosis and open resection [10].

Although the standard of care for the treatment of OO is image-guided RF ablation, in subchondral lesions, as in our case, cartilage degeneration is a possible complication and needs attention, especially in weight-bearing joints [5,19]. A distance of 1 mm or more from the cartilage is considered safe, and articular cartilage appears to be relatively tolerant to thermal injury induced by short-term heating [20]. For most patellar OO, open resection was the chosen treatment, while Bavaneh et al. applied mosaicplasty technique after open resection for the cartilage defect [7,10,13]. On the other hand, Franceschi et al. described a CT-guided arthroscopic resection of a subchondral patellar OO and Chillemi et al. described a percutaneous CT-guided drilling of the nidus with good results [11,12]. Our patient was successfully treated with the method of RF ablation under CT guidance (Figure 6) without any cartilage complications as implied by the complete resolution of the symptoms and absence of complaints during follow-up.

## Conclusions

Although a rare entity, OO of the patella with its atypical clinical features should be included in the differential diagnosis of persistent AKP in the young adult. Especially in adolescents presenting with long-standing non-specific complaints around the knee joint with non-conclusive MRI findings, CT scan seems a reasonable step in the diagnostic procedure. High clinical suspicion for this rare lesion is necessary for the orthopedist to avoid delay in diagnosis and irrelevant procedures for the patient. Percutaneous CT ablation seems a safe technique for the treatment of patellar OO, although attention is needed to avoid any cartilage damage.
